# Radiosurgical Ablation of Solitary Trichoepithelioma

**DOI:** 10.4103/0974-2077.41156

**Published:** 2008-01

**Authors:** H C Hanumanthiah

**Affiliations:** *Consultant Dermatologist, Skin Care Clinic, No. 99, 3^rd^ Main, KEB Layout, BTM 1^st^ Stage, Bangalore, Karnataka, India*

**Keywords:** Trichoepithelioma, radiofrequency, ablation, solitary

## Abstract

Trichoepithelioma presents a challenge for management in view of its location. We describe the use of radiofrequency ablation in the management of this condition.

## INTRODUCTION

Trichoepitheliomas are well differentiated benign follicular tumours that are particularly important because they may be clinically and histologically confused with basal cell carcinomas (BCCs). The recognised clinicopathalogical types are:[1]

- Solitary trichoepithelioma
- Multiple trichoepitheliomas
- Desmoplastic trichoepithelioma
- Giant solitary trichoepithelioma

No conservative treatment is available. Ablative intervention is the only management if required for cosmetic reasons.[2]

## CASE REPORT

**Figure 1:**
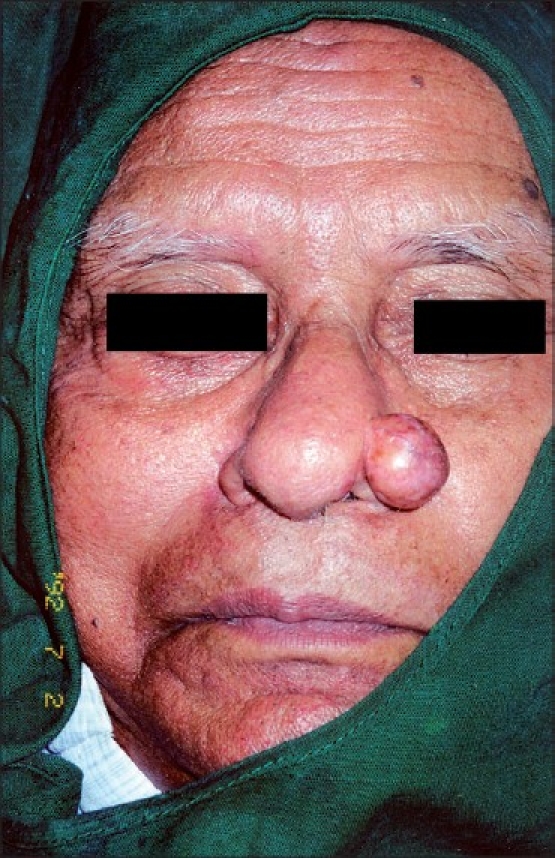
Skin colored nodule on the left ala nasi

A 71-year-old man presented with asymptomatic skin colored nodule, measuring around 2 cm over the left ala nasi of 5 years duration [Figure 1]. Differential diagnosis of keratoacanthoma, dermatofibroma, trichoepithelioma (solitary) and BCC was considered.

**Figure 2:**
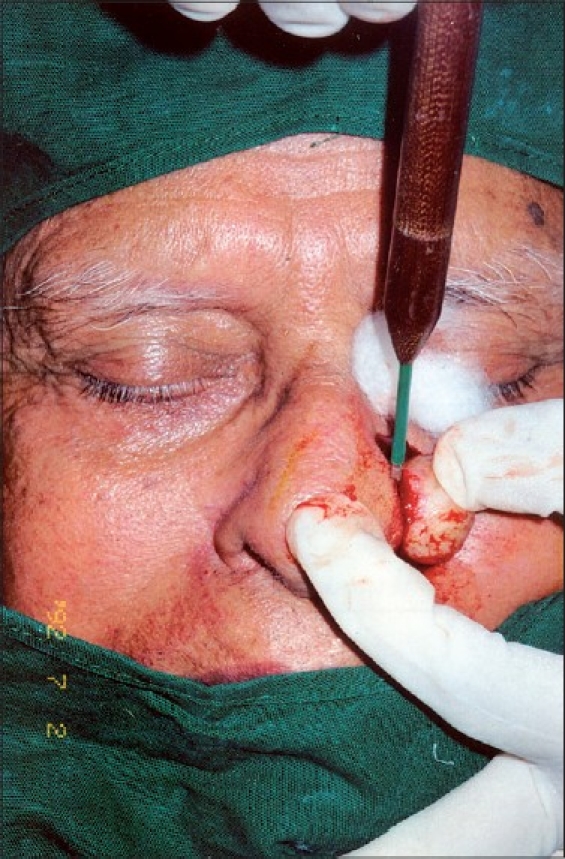
Nodule being removed by radiofrequency, cutting mode

Under 1% local anaesthesia with adrenaline the lesion was excised by cutting mode of radio frequency (RF) surgery [Figure 2].

The bleeding was controlled by putting figure of eight suture with 3-0 plain catgut followed by dressing for a day. Topical and oral antibiotics and anti-inflammatory drugs were advised for 7 days.

Excision biopsy report came as solitary trichoepithelioma.

## DISCUSSION

Solitary trichoepithelioma is a slowly growing epithelial tumour. There is no genetic association. It presents as a single skin colored nodule, usually on the adult face. Excision is the treatment of choice. The radiosurgery was used for its accurate removal of lesion with minimal bleeding without destroying underlying structures of ala nasi.

This case is being reported for its rarity and for successful treatment by cutting mode of RF surgery without damaging the underlying cartilaginous structures of ala nasi.
